# Gaze Synchrony between Mothers with Mood Disorders and Their Infants: Maternal Emotion Dysregulation Matters

**DOI:** 10.1371/journal.pone.0144417

**Published:** 2015-12-14

**Authors:** Annett Lotzin, Georg Romer, Julia Schiborr, Berit Noga, Michael Schulte-Markwort, Brigitte Ramsauer

**Affiliations:** 1 University Medical Center Hamburg-Eppendorf, Department of Child and Adolescent Psychiatry, Psychotherapy and Psychosomatics, Hamburg, Germany; 2 Department of Child and Adolescent Psychiatry, Psychosomatics and Psychotherapy, University Hospital Muenster, Muenster, Germany; University of Rennes-1, FRANCE

## Abstract

A lowered and heightened synchrony between the mother’s and infant’s nonverbal behavior predicts adverse infant development. We know that maternal depressive symptoms predict lowered and heightened mother-infant gaze synchrony, but it is unclear whether maternal emotion dysregulation is related to mother-infant gaze synchrony. This cross-sectional study examined whether maternal emotion dysregulation in mothers with mood disorders is significantly related to mother-infant gaze synchrony. We also tested whether maternal emotion dysregulation is relatively more important than maternal depressive symptoms in predicting mother-infant gaze synchrony, and whether maternal emotion dysregulation mediates the relation between maternal depressive symptoms and mother-infant gaze synchrony. We observed 68 mothers and their 4- to 9-month-old infants in the Still-Face paradigm during two play interactions, before and after social stress was induced. The mothers’ and infants’ gaze behaviors were coded using microanalysis with the Maternal Regulatory Scoring System and Infant Regulatory Scoring System, respectively. The degree of mother-infant gaze synchrony was computed using time-series analysis. Maternal emotion dysregulation was measured by the Difficulties in Emotion Regulation Scale; depressive symptoms were assessed using the Beck Depression Inventory. Greater maternal emotion dysregulation was significantly related to heightened mother-infant gaze synchrony. The overall effect of maternal emotion dysregulation on mother-infant gaze synchrony was relatively more important than the effect of maternal depressive symptoms in the five tested models. Maternal emotion dysregulation fully mediated the relation between maternal depressive symptoms and mother-infant gaze synchrony. Our findings suggest that the effect of the mother’s depressive symptoms on the mother-infant gaze synchrony may be mediated by the mother’s emotion dysregulation.

## Introduction

The eyes fascinate us. From the day we are born, we are strongly sensitive to the gaze of others [1, 2]. The gaze of our interaction partners attract us because it conveys the partners’ focus of attention [3, 4], willingness for interaction [4], and emotional states and intentions [5, 6]. Additionally, the gaze of others regulates our arousal and emotions. When a person gazes directly at us, we may become aroused and feel a certain emotion. At the same time, we influence the arousal and emotions of this person by looking into the person’s eyes, looking nearby, or averting the gaze.

Coordinating our gaze with the gaze of our interaction partner occurs at latest during the third month of life [7–9]. For infants, gaze coordination is particularly important because it offers learning opportunities [7–9]. The shared attention between mother and infant helps the infant to understand the meaning of language, intentions and emotions of others [10–13]. In time, the mother-infant coordination of gaze enables the infant to repeatedly experience contingent behavior through which the infant develops expectations concerning “what comes next” in the social world [14]. Thus, the coordination of gaze between mother and infant affects the infant’s cognitive [5], language [12, 15], and socio-emotional development [16, 17].

An integral feature of the temporal coordination between mother and infant is synchrony [18], i.e., the degree of the *temporal coordination* of behaviors between interaction partners [19], which is determined by two dimensions, behavior and time. According to an “optimal midrange model of self- and interactive regulation” [11], a well-organized and flexible interaction between mother and infant is characterized by a moderate degree of synchrony of their attentive-affective behavior, ranging from .03 to .14 after controlling for the rhythm within each partner’s behavior [20–23]. Conversely, both lowered and heightened mother-infant synchrony characterizes maladaptive mother-infant interaction [11, 24], which interferes with the infant’s socio-emotional development [24].

Maternal depressive symptoms are related to both lowered and heightened mother-infant gaze synchrony. Depression is the most frequent mental disorder in the postpartum period, which affects 13 out of 100 mothers after birth [25]. Mothers with depression are unresponsive or intrusive towards their infant (for reviews see [26, 27]). Infants of mothers with depression engage less with objects and in social interaction and develop less optimal self-regulation abilities than infants of mothers without depression [28–30]. Over a longer period, children of mothers with depression demonstrate unfavorable cognitive, behavioral and health-related outcomes until their adolescence [31, 32].

A number of studies demonstrated in non-clinical samples that maternal more severe depressive symptoms [33], greater distress [34] and greater self-criticism [35] were related to lowered mother-infant gaze synchrony. Conversely, greater feelings of dependency [35] were related to heightened mother-infant gaze synchrony. Such deviances in the synchrony between the mother’s and infant’s behavior have long-lasting negative effects on the infant, including lower self-regulation abilities, unsecure attachment, less competence in symbol use and less empathy towards others [16, 24, 36].

Why maternal psychopathology, such as depressive symptoms, predict deviant mother-infant synchrony remains unclear. The psychopathological symptoms might be related to dysfunctions in the mother’s ability to regulate her emotions, which may affect her ability to regulate gaze during mother-infant interaction. The ability to regulate emotions refers to processes that modulate emotions for appropriately responding to environmental demands [37–39]. Such processes involve the awareness and understanding of emotions, the acceptance of emotions, and the ability to control impulsive behaviors when experiencing negative emotions to meet individual goals [38].

Emotion regulation is impaired in individuals with mental disorders, including mood disorders [40–42]. During mother-infant interaction, depressed mothers face the challenge of simultaneously regulating their negative emotions and the emotional behavior of their infants. The maternal ability to regulate negative emotions may explain the effect of the maternal depressive symptoms on the observed mother-infant gaze synchrony. However, this assumption has not been tested.

Additional gaps in mother-infant gaze synchrony research must be explored. First, researchers only investigated the relationships between maternal depression-related variables and mother-infant gaze synchrony in non-clinical samples [24, 33–35], in which the psychopathology variance is restricted. Second, the studies on mother-infant gaze synchrony should consider that maternal depressive symptoms co-occur with anxiety symptoms [43–45]. The likely impact of co-morbid anxiety symptoms in mothers with depression on mother-infant interaction has been overlooked [46]. Third, gaze synchrony was measured during unstructured interaction which demand lower regulation abilities [24, 33–35]. Mother-infant gaze synchrony might remain intact in unstructured mother-infant interactions, although it might deviate in interactions with higher regulatory demands, for example when mothers are asked to fulfill a certain task.

In this study, we tested the effects of maternal emotion dysregulation and depressive symptoms on mother-infant gaze synchrony in a sample of mothers with mood disorders, after controlling for maternal anxiety symptoms, infant gender and infant age. We micro-analytically measured the mother’s and infant’s gaze in two face-to-face play interactions using the Still-Face paradigm, before and after stress had been induced by the non-responsiveness of the mother. Mother-infant gaze synchrony was calculated using time-series analysis. We hypothesized that (1) maternal self-reported emotion dysregulation is significantly positively related to mother-infant gaze synchrony; (2) the play condition significantly moderates the relationship between maternal emotion dysregulation and mother-infant gaze synchrony: maternal emotion dysregulation is more strongly related to mother-infant gaze synchrony in the reunion play than in the initial play; (3) maternal emotion dysregulation is relatively more important than maternal depressive symptoms in predicting mother-infant gaze synchrony; and (4) maternal emotion dysregulation mediates the relation between maternal depressive symptoms and mother-infant gaze synchrony.

## Materials and Methods

This cross-sectional study is part of a larger study [47] that was approved by the local Ethics Committee of the Medical Board of Hamburg, Germany (reference number PV3269).

### Sample characteristics

We included dyads if (1) the mother was diagnosed with a mood disorder, current episode depressed, according to the Diagnostic and Statistical Manual of Mental Disorders-IV (DSM-IV, [48]); (2) the mother provided informed consent to participate in the study; (3) the mother was a fluent speaker of German; and (4) the infant was aged 4 to 9 months. We excluded dyads if (1) the mother was diagnosed with a psychosis, primary substance abuse or intellectual impairments, or reported an acute psychiatric crisis; or (2) the infant was diagnosed with a pervasive developmental disorder. Additional information regarding study methodology is provided elsewhere [47].

The mothers were between 20 and 44 years old, with a mean age of 32 years. The majority of the mothers were unmarried, but lived with their partner in one household (Table 1).

**Table 1:** Characteristics of Mothers with Mood Disorders and Their Infants (*N* = 68).

Characteristic	*M*	*SD*
Infant age (months)	6.3	1.8
Week of pregnancy at birth	39.4	1.9
Maternal age (years)	32.2	5.4
Maternal education (years)	15.2	3.0
BDI score	20.2	10.8
DERS total score	106.6	25.5
Infant gender	f	%
Male	39	57.4
Female	29	42.7
Parity		
Firstborn	62	83.8
Second-born	10	13.5
Third-born	2	2.7
Maternal ethnic background		
European Caucasian	67	98.5
African	1	1.5
Marital status		
Never married	40	58.8
Married	27	39.7
Divorced	1	1.5
Living status with partner		
No partner	10	14.7
Living together	52	76.5
Living apart	6	8.8
Monthly household income (Euro)^a^		
≤ 1000	9	13.2
1001–2000	13	19.1
2001–3000	24	35.3
≥ 3001	18	26.5
Maternal psychiatric medication		
Yes	28	41.2
No	40	58.8

^a^ *n* = 64.

The family’s socioeconomic status ranged from lower to upper middle-class. The mothers’ years of education were unrelated to mother-infant gaze synchrony (β = -0.03, 95% CI [-0.04, 0.10], *t*(67.2) = 0.81, *p* = .423). The infants’ ages ranged from 4 to 9 months; on average, the infants were 6 months old. Approximately 50% of the infants were male. Infants were born after 39 weeks and 3 days of pregnancy, on average. Most infants were firstborn.

All mothers were diagnosed with a DSM-IV Axis I mood disorder (88.2% major depressive disorder; 4.4% dysthymia; 4.4% bipolar disorder, currently depressed; 2.9% adjustment disorder with depressed mood). Co-morbidity was common in our sample: 55.9% of the mothers suffered from at least one co-morbid anxiety disorder (14.7% posttraumatic stress disorder; 14.7% social phobia; 10.3% panic disorder with agoraphobia; 8.8% panic disorder without agoraphobia; 4.4% generalized anxiety disorder; 4.4% obsessive-compulsive disorder; and 1.5% specific phobia).

According to the Beck Depression Inventory (BDI, [49]), the mother’s mean score of depressive symptoms (M = 20.2, range 1 to 46) was indicative of clinically relevant depressive symptoms. At the time of the behavioral assessment, 25.0% of the patients reported minimal depressive symptoms, 14.7% reported mild-to-moderate depressive symptoms, and 60.3% reported severe depressive symptoms.

The majority (58.8%) of the mothers received no medical treatment for their mental disorder, often because mothers were breastfeeding. One third (35.3%) of the mothers reported that they received antidepressants (32.4% received selective serotonin reuptake inhibitors, or serotonin-norepinephrine reuptake inhibitors; 1.4% received tetracyclic antidepressants; and 1.5% received other antidepressants). One mother (1.5%) was treated with benzodiazepine, and three mothers (4.4%) were treated with atypical neuroleptics. Whether mothers took medication or not was unrelated to mother-infant gaze synchrony (β = -0.23, 95% CI [-0.65, 0.19], *t*(65.9) = -1.07, *p* = .287).

The mothers reported a high severity of emotion dysregulation according to the Difficulties in Emotion Regulation Scale (DERS, [38]) total score on average (M = 106.6, range 54 to 174). A score of ≥ 103 was reported by 50% of the mothers, whereas such high levels of emotion dysregulation were reported only by 10% of the mothers in a nonclinical female standardization sample [38].

### Procedure

Mothers and their infants were recruited from a psychiatric mother-infant outpatient unit that offers mother-infant treatment for mentally ill mothers and their infants at the University Medical Center of Hamburg, Germany. Most of the mothers were referred to the outpatient unit from other medical or mental health services of Hamburg, an urban city with 1.8 million inhabitants. Mother-infant dyads were recruited and assessed from September 2009 to February 2013.

Mothers and their infants potentially eligible for inclusion in the study were contacted by an experienced clinician, informed about the study and invited to participate. Both custodial parents had to provide informed consent for their infant to participate in the study. After both parents had signed the informed consent, the mother was invited to the mother-infant outpatient unit to participate in the Structured Clinical Interview I for DSM-IV (SCID-I, [50]), which was performed by trained research assistants. Mothers filled out questionnaires that assessed demographic characteristics, clinical symptoms and difficulties in emotion regulation. After completion, the research assistant determined whether the mother and infant met the eligibility criteria of the study.

A behavioral observation in the mother-infant research unit was requested when the infant was approximately 6 months old. Mother-infant interaction was filmed in the Still-Face paradigm [51]. Four cameras captured the mother and infant from four different directions using a hard disk recording system.

### Measures

#### Maternal clinical characteristics

The SCID-I [50] is an established method for assessing DSM-IV diagnoses, for which sufficient reliability and validity have been reported [52–54]. The Beck Depression Inventory (BDI, [49]) is a 21-item, self-report inventory that measures depressive symptoms that occurred during the last week. The items are scored from 0 to 3 and summed to obtain a total depression score (ranging from 0 to 63). The cut-offs for “no to minimal” (0–10), “mild to moderate” (11–17) and “clinically relevant” depressive symptoms (18–63) can be derived. The psychometric properties of the BDI have been synthesized in a meta-analysis [55]; the authors reported a mean internal consistency (Cronbach's α) of .86 for psychiatric patients, evidence for concurrent validity that is based on high correlations using clinical ratings and the Hamilton Psychiatric Rating Scale for Depression, and evidence for discriminating depression from anxiety. In our sample of mothers with mood disorders, we found a mean internal consistency of the BDI total score of Cronbach's α = .91.

Because approximately half of the mothers in our study suffered from a comorbid anxiety disorder, we controlled our analysis for maternal anxiety symptoms. Mothers evaluated their current severity of anxiety using the Anxiety subscale of the German version of the Symptom Checklist-90-Revised (SCL-90-R, [56]), which is a widely used self-report inventory for assessing nine dimensions of psychopathology. The anxiety symptoms covered by the SCL-90-R are rated within the last week using 10 five-point Likert scales ranging from 0 (“not at all”) to 4 (“extremely”). The SCL-90-R anxiety score is calculated as the average score of the 10 items. Studies demonstrated convergent, divergent and criterion validity [56–58]. The SCL-90-R anxiety subscale discriminates from the SCL-90-R depression subscale in anxiety [59, 60] and depression [60] samples. The ten items of the SCL-90-R Anxiety subscale showed good internal consistency (Cronbach's α = .87).

#### Emotion dysregulation

Maternal reported emotion dysregulation was assessed using the Difficulties in Emotion Regulation Scale (DERS, [38]). This 36-item questionnaire measures clinically relevant difficulties in emotional regulation. The participants rate on a five-point Likert scale (1 = “almost never” to 5 = “almost always”) how frequently each statement applies to them. The DERS total score (range 36 to 180) represents the overall difficulties in emotion regulation, which is based on six subscale scores (Nonacceptance of emotional responses, Difficulties to engage in goal-directed behavior, Difficulties in controlling impulsive behaviors, Lack of emotional awareness, Limited access to emotion regulation strategies, and Lack of emotional identification or clarity). Higher scores indicate greater emotion dysregulation.

The DERS has been used in clinical populations with mood disorders [61] and anxiety disorders [62–64]. Convergent validity with other self-reports of emotion regulation [61, 65, 66], and convergent validity with behavioral [66] and physiological [67] measures of emotion regulation were reported. The internal consistency of the DERS total score was Cronbach's α = .87 in our sample.

#### Observation of mother-infant interaction

Mother-infant interaction was observed in the Still-Face paradigm [51]. This standardized experiment was designed to observe the face-to-face interaction between a mother and her infant 3 to 9 months of age. The mother places her infant in an infant chair and directly faces her infant. The paradigm consists of three consecutive episodes: an initial play condition of a spontaneous interaction between the mother and the infant (3 min); a “still-face” episode, in which the mother is non-responsive to her infant (1 min); and a reunion play condition, in which the mother and infant reengage in a spontaneous interaction (3 min). The maternal unresponsiveness during the still-face episode reliably evokes typical stress reactions in infants, such as reduced positive emotions, increased negative emotions and gaze aversion, and increased cortisol levels [68, 69]. These stress reactions are carried over to the reunion play condition [69].

#### Coding of gaze behavior

The first 3 minutes of the initial play and the reunion play of the Still-Face paradigm were coded (some episodes were slightly longer than 3 minutes, for example because the mothers did not immediately notice the knocking sound that signaled the start of the still-face episode). The mothers’ and infants’ behavior were micro-coded 25 times per second (every 40 ms) using a software system for behavioral coding (The Observer XT 11.0, Noldus Information Technology, Wageningen, Netherlands), which resulted in two time-series of 4,500 data points for each mother and each infant.

The infants’ and the mothers’ gaze behavior were coded with the Maternal Regulatory Scoring System (MRSS, [70]) and the Infant Regulatory Scoring System (IRSS, [71]). The MRSS and IRSS are established coding systems of nonverbal face-to-face interaction behavior between caregivers and infants [72]. The two complementary coding systems were derived from Tronick's Monadic Phase Scoring System [73]. The MRSS was designed to separately code six modalities of the parent’s behavior (Direction of gaze, Caregiving behavior, Proximity to infant, Vocalizations, Touch, and Eliciting behavior). The IRSS can be used to code behavior of infants younger than one year on five dimensions (Direction of gaze, Gestures, Vocalization, Self-Comfort, Distance, and Autonomic Indicators). Scoring of video-recorded interactions is done micro-analytically. Based on the gaze coding rules of the MRSS and IRSS, infants’ and mothers’ gaze behavior were coded using four mutually exclusive codes: 1 = “Gaze at the partner’s face” 2 = “Gaze at object” 3 = “Gaze away” and 99 = “Unscorable”. “Gaze at the partner’s face” was coded when the mother or infant gazed at the partner’s eyes or upper face. “Gaze at object” was scored when the mother or infant gazed at a proximal object between the mother and infant, e.g., the partner’s body or the infant’s seat. “Gaze away” was coded when the mother or infant gazed away from the partner’s face *and* did not gaze at a proximal object or the mother closed her eyes or the infant closed his eyes. “Unscorable” was coded when the eyes were not visible in any of the videos shot by the four cameras.

The inter-rater reliability of the MRSS [74–77] and IRSS [72, 75, 76, 78, 79] can be regarded as excellent. Evidence for their convergent validity using the AFFEX system has been reported [4, 77, 80]. The IRSS discriminates between mothers with and without depression [72].

Two raters, i.e., a doctoral student and a researcher, were trained extensively for 30 hours over a 3-month period to a reliability level of Κ ≥ .80. The doctoral student, who was blinded to other data in the study, coded the videos. Training included the coding of parent-infant interaction videos (that were not used in this study) according to the scoring rules of the coding manual. The coding order of the videos was randomized. In total, 272 time series of gaze behavior were coded (68 mothers and 68 infants in two play conditions).

To assess the inter-rater reliabilities, the infant’s and mother’s behavior of 100 (33.8%) randomly selected time series were independently coded by the second coder. The Kappa coefficients (time window 40 ms) indicated excellent inter-rater reliability for the coding of the mother’s (initial play Κ = .95; reunion play Κ = .95) and the infant’s gaze behavior (initial play Κ = .96; reunion play Κ = .97).

#### Data preparation and analysis

Based on an a priori power analysis using PASS version 11 [81], we estimated a sample size of 71 dyads to achieve 80% power to detect an R² of .10. We used an F-Test with a .05 alpha level of significance and attributed 80% power to two independent variables (emotion dysregulation and play condition), which were adjusted for three control variables with an R² of .20.

Missing data from the questionnaire assessments were rare (0.1%) because the investigators ensured that the questionnaires were entirely completed at every visit. Uncodable behavior of the mothers or infants rarely occurred (mother’s gaze, 0.8%; infant’s gaze, 2.1%). Missing data of the independent variables were imputed using the Expectation-Maximization (EM) algorithm of IBM SPSS Statistics Version 21. In the reunion episode, the mother-infant gaze synchrony could not be calculated for two observations, because the gaze behavior of mother and infant was constant. For these two observations, we did not impute the missing synchrony data.

To compare with previous studies [33, 35, 82], the gaze scores were rounded to whole seconds according to the scoring rules of the MRSS [70] and IRSS [71] coding manuals, resulting in a time series of 180 gaze states for each observation of the mother or infant. Mother-infant gaze synchrony was computed separately for individual dyads using time-series analysis [19]. Before calculating the synchrony between the two time-series of mother-infant gaze behavior, the time series were inspected for stationarity (consistency of mean and variance across time) by using linear regression analysis (gaze behavior was regressed on time). If the stationary condition was violated (this was the case for 1.5% of all time series) the time series was differenced. The autocorrelation in each time series was estimated using autoregressive integrated moving average (ARIMA) models. The autocorrelation was partialled out of each time series to control for the cyclicity within an individual’s behavior [19]. Cross-correlation functions (CCFs) were computed for each dyad using the two series of residuals from the ARIMA models. The largest cross-correlation between the two time series found at any lag indexed the synchrony between the two time-series. This measure of synchrony ranged from 0 (no lagged associations between the two time series) to 1 (perfect match between the time series).

The lead-lag relationships between the mother’s and the infant’s behavior was assessed to determine whether mother or infant (or both) synchronized with the partner’s gaze behavior. A significant peak on the CCF plot at a positive lag indicated mother synchrony with infant (the mother responded to changes in the infant’s behavior). A significant peak on the CFF plot at a negative lag indicated infant synchrony with mother (the infant responded to changes in the mother’s behavior). Mutual synchrony (both partners responded to changes in each other’s behavior) was assumed when significant peaks on the CCF could be found at a positive and at a negative lag.

Infant age, infant gender, and maternal anxiety symptoms were included in our analyses as potential confounders, because evidence exists that these variables may be related to mother-infant synchrony [83]. To test whether the potential confounders (infant age, infant gender, and maternal anxiety symptoms), maternal depressive symptoms, maternal emotion dysregulation and the play condition (initial vs. reunion play condition) explained the variance of the mother-infant gaze synchrony, we fitted a Multilevel Random Coefficient Model (MRCM). We used IBM SPSS Statistics Version 21 to calculate the MCRM. We fitted five models, each including one random effect for the intercept to control for the clustering within subjects across the two measurement points. The fixed effects predictors were stepwise included in the models to determine whether the independent variable(s) would predict mother-infant gaze synchrony.

In Model 0, only the random intercept was included in the model to test whether the random effect explained the variance between dyads. In Model 1, the play condition (initial vs. reunion play) was added as a fixed effect. The variables, infant gender, infant age, maternal anxiety symptoms and maternal depressive symptoms, were entered into Model 2 to examine whether these variables predicted mother-infant gaze synchrony. Maternal emotion dysregulation was included as an additional predictor in Model 3 to address our hypothesis that maternal emotion dysregulation is significantly related to mother-infant gaze synchrony, after controlling for infant gender, infant age, maternal anxiety and maternal depressive symptoms. In Model 4, the interaction term of maternal emotion dysregulation and the play condition was entered as a fixed cross-level effect in the model to test our hypothesis that the play condition moderates the effect of maternal emotion dysregulation on mother-infant gaze synchrony.

To determine the relative importance of maternal emotion dysregulation and maternal depressive symptoms to predict mother-infant gaze synchrony in the MRCM, we used information-theoretic model comparisons [84]. In a first step, we selected the predictors that were significantly related to mother-infant gaze synchrony in at least one of the five MRCM. All possible combinations of these variables were tested in separate models to predict mother-infant gaze synchrony. The number of all possible combinations can be calculated by 2^(n of significant predictors)^ -1. For example, if the variables A, B and C significantly predicted mother-infant gaze synchrony, seven separate models (2^3^–1 = 7) with the following combinations of predictors would be tested: A; B; C; AB; AC; BC; ABC. This entire set of models of possible combinations of the variables were tested to predict mother-infant gaze synchrony. The relative influence of the explanatory predictors were assessed by summing the Akaike weights [84], ∑ω, of each model in which a predictor occurred.

To examine whether maternal emotion dysregulation would mediate the relation between maternal depressive symptoms and mother-infant gaze synchrony, a mediation analysis [85] using MRCM with IBM SPSS Statistics Version 21 was conducted. Infant age, infant gender and maternal anxiety symptoms were included in the mediation analysis as potential confounders. The MRCM included a random intercept to control for the clustering within dyads. According to Baron & Kenny [86], four conditions confirm a mediation model: condition I: the independent variable (maternal depressive symptoms) must be significantly related to the mediator (maternal emotion dysregulation); condition II: the independent variable (maternal depressive symptoms) must be significantly related to the dependent variable (mother-infant gaze synchrony); condition III: the mediator (maternal emotion dysregulation) must be significantly related to the dependent variable (mother-infant gaze synchrony); condition IV: the effect of the independent variable (maternal depressive symptoms) on the dependent variable (mother-infant gaze synchrony) must significantly decrease when the mediator (maternal emotion dysregulation) has been partialled out.

## Results

**Fig 1:**
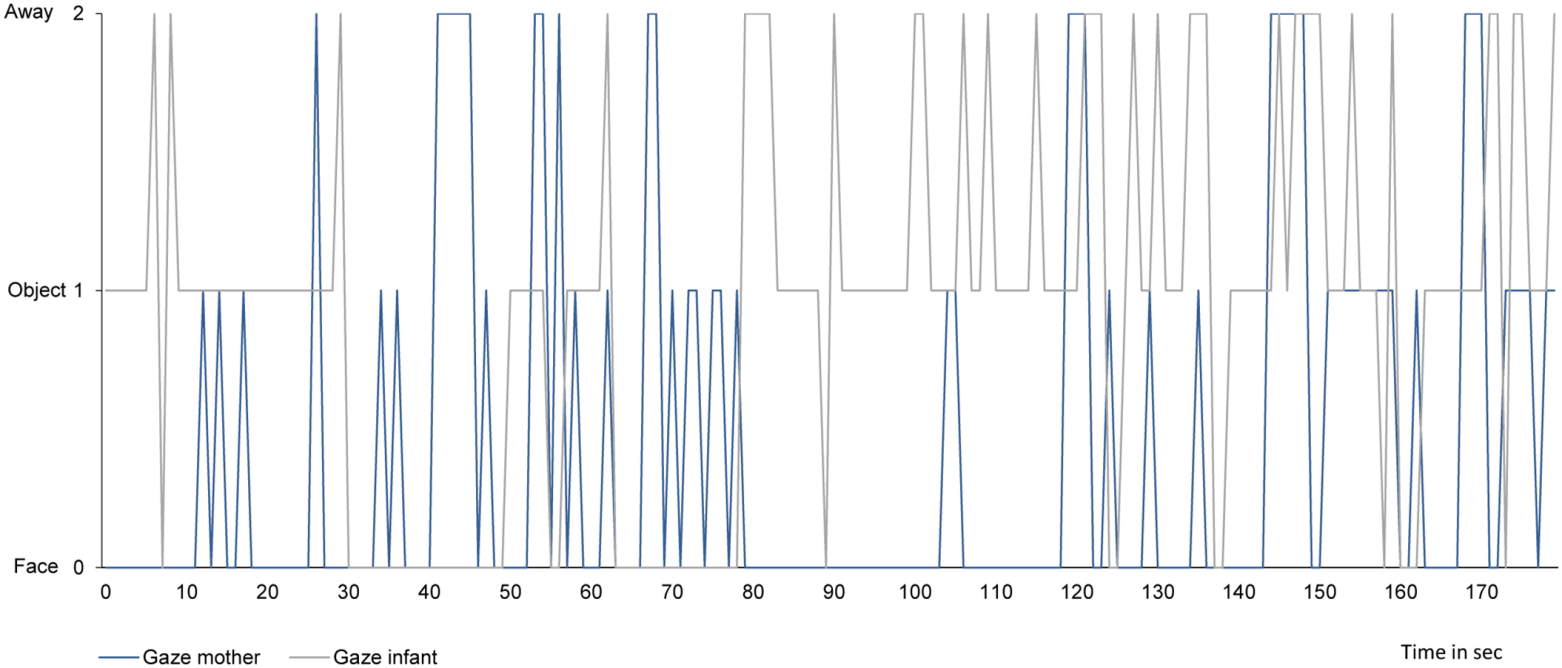
Gaze behavior of one mother-infant dyad during a 3-minute play with lower mother-infant gaze synchrony. Face = gaze at the partner’s face. Object = gaze at proximal object between mother and infant. Away = gaze away from interaction partner.

After screening 118 mothers with infants, 68 mother-infant dyads were included in this study. Fig 1 illustrates the gaze behavior of one mother-infant dyad with lower mother-infant gaze synchrony.

**Fig 2:**
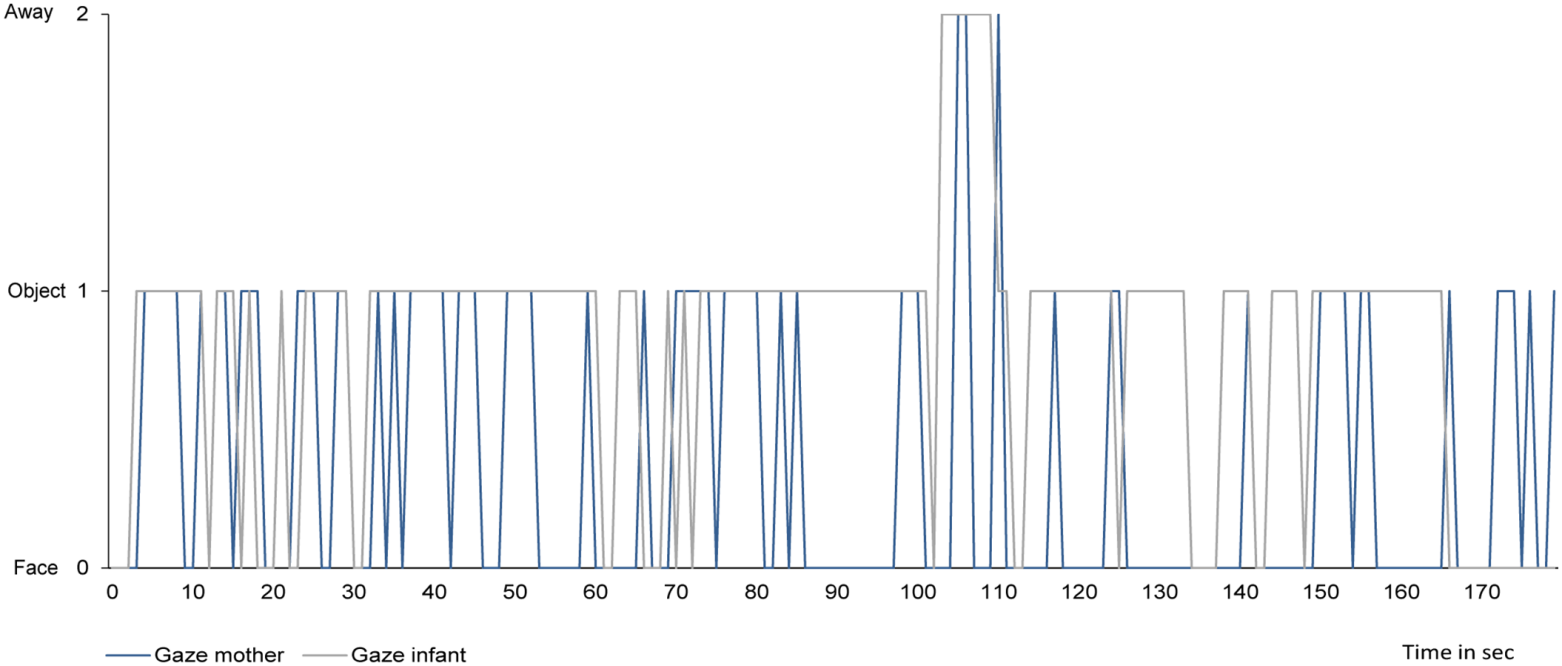
Gaze behavior of one mother-infant dyad during a 3-minute play with higher mother-infant gaze synchrony. Face = gaze at the partner’s face. Object = gaze at proximal object between mother and infant. Away = gaze away from interaction partner.

Fig 2 illustrates the gaze behavior of one mother-infant dyad with higher mother-infant gaze synchrony.

On average, mothers and infants gazed approximately 5 times per minute at the face of the interaction partner in the initial and reunion play (Table 2). Mothers gazed away 2 times per minute in the initial and reunion play, whereas infants gazed away 3 times per minute in the initial play and 4 times per minute in the reunion play, on average. Mothers and infants spent their greatest proportion of time in gazing at the interaction partner’s face.

**Table 2:** Gaze Behavior of Mothers and Infants and Mother-Infant Gaze Synchrony in the Initial Play and the Reunion Play (*N* = 68).

Gaze variable	Initial play		Reunion play	
Gaze frequency per min	*M*	*SD*	*M*	*SD*
Mother				
Face	5.0	2.9	5.1	2.8
Object	3.1	2.3	3.1	2.4
Away	2.0	2.1	1.9	1.9
Infant				
Face	4.7	1.7	4.7	2.0
Object	3.5	1.9	2.8	2.1
Away	3.2	2.0	3.7	2.3
Gaze percent of time				
Mother				
Face	84.4	11.5	85.0	10.9
Object	10.4	9.2	9.8	9.0
Away	5.3	5.8	5.2	5.4
Infant				
Face	51.9	23.8	43.6	25.1
Object	26.9	20.0	22.0	19.9
Away	21.3	19.0	34.3	28.9
Gaze synchrony^a^	.11	.05	.15	.08

^a^Gaze synchrony between mother and infant as measured by time-series analysis.

In the initial play, mother-infant gaze synchrony ranged from .00 to .30. A low degree of synchrony (range .000–.030) was shown by two dyads; a moderate degree of synchrony (range .031–.140) was observed in 51 dyads; and a high degree (range .141–.300) was observed in 15 dyads. The mother-infant gaze synchrony increased from the initial play to the reunion play. In the reunion play, mother-infant synchrony ranged from .00 to .45. A low degree of synchrony (range .000–.030) was shown by one dyad; a moderate degree of synchrony (range .031–.140) was observed in 38 dyads. A high degree of synchrony (range .141–.450) was observed in 27 dyads.

Maternal depressive symptoms and maternal emotion dysregulation were significantly related to mother-infant gaze synchrony in the initial and the reunion play (Table 3).

**Table 3:** Intercorrelations between the Predictor Variables and Mother-Infant Gaze Synchrony in the Initial Play and the Reunion Play of the Still-Face Paradigm (*N* = 68).

	Synchrony initial play^a^	Synchrony reunion play^a^	Infant female	Infant age	Depressive symptoms^b^	Anxiety symptoms^c^
Synchrony reunion^a^	.50***					
Infant female	-.24*	-.13				
Infant age	.07	.08	-.30*			
Depressive symptoms^b^	.34**	.31*	-.06	.15		
Anxiety symptoms^c^	.25*	.23	-.12	.12	.55***	
Emotion dysregulation^d^	.37**	.39***	-.11	.14	.75***	.48***

*Note*. Pearson correlations were calculated for the association between two continuous variables, point-biserial correlations were calculated for the association between a continuous and a dichotomous variable.

^a^Gaze synchrony between mother and infant as measured by time-series analysis.

^b^Beck Depression Inventory.

^c^Anxiety score of the Symptom Checklist-90-Revised.

^d^Difficulties in Emotion Regulation Scale.

*p ≤ .05.

**p ≤ .01.

***p ≤ .001.

**Fig 3:**
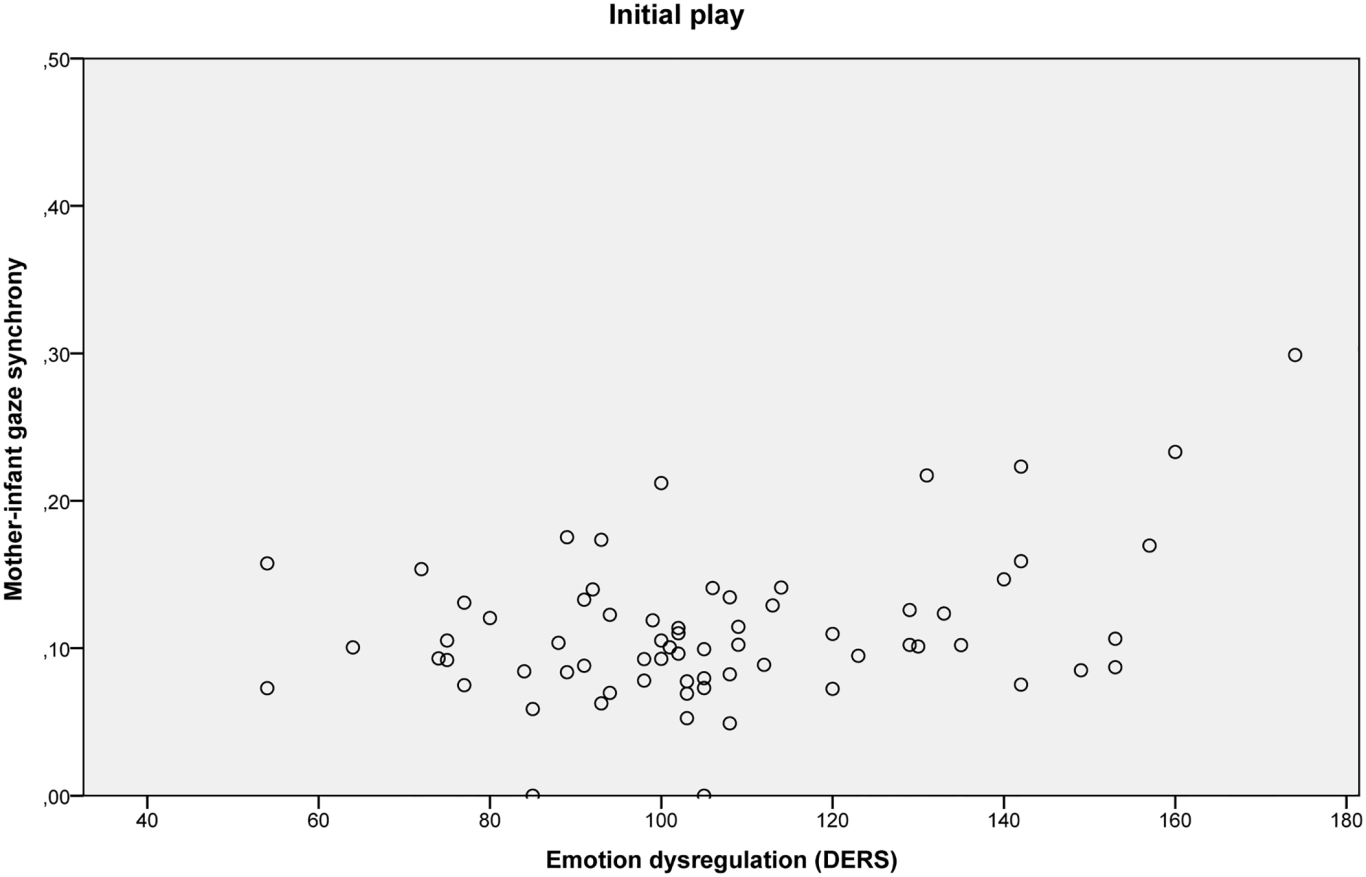
Scatter plot of the mothers’ emotion dysregulation scores and mother-infant gaze synchrony scores in the initial play.

The intercorrelations between maternal depressive symptoms, maternal anxiety symptoms and maternal emotion dysregulation were moderate to high. The Variance Inflation Factors (VIFs, [87]) of these variables were lower than 10 (depressive symptoms VIF = 2.65, anxiety symptoms VIF = 1.46, emotion dysregulation VIF = 2.49), indicating no potential problems with multicollinearity. Fig 3 presents a scatter plot of the mothers’ emotion dysregulation scores versus mother-infant gaze synchrony scores in the initial play.

**Fig 4:**
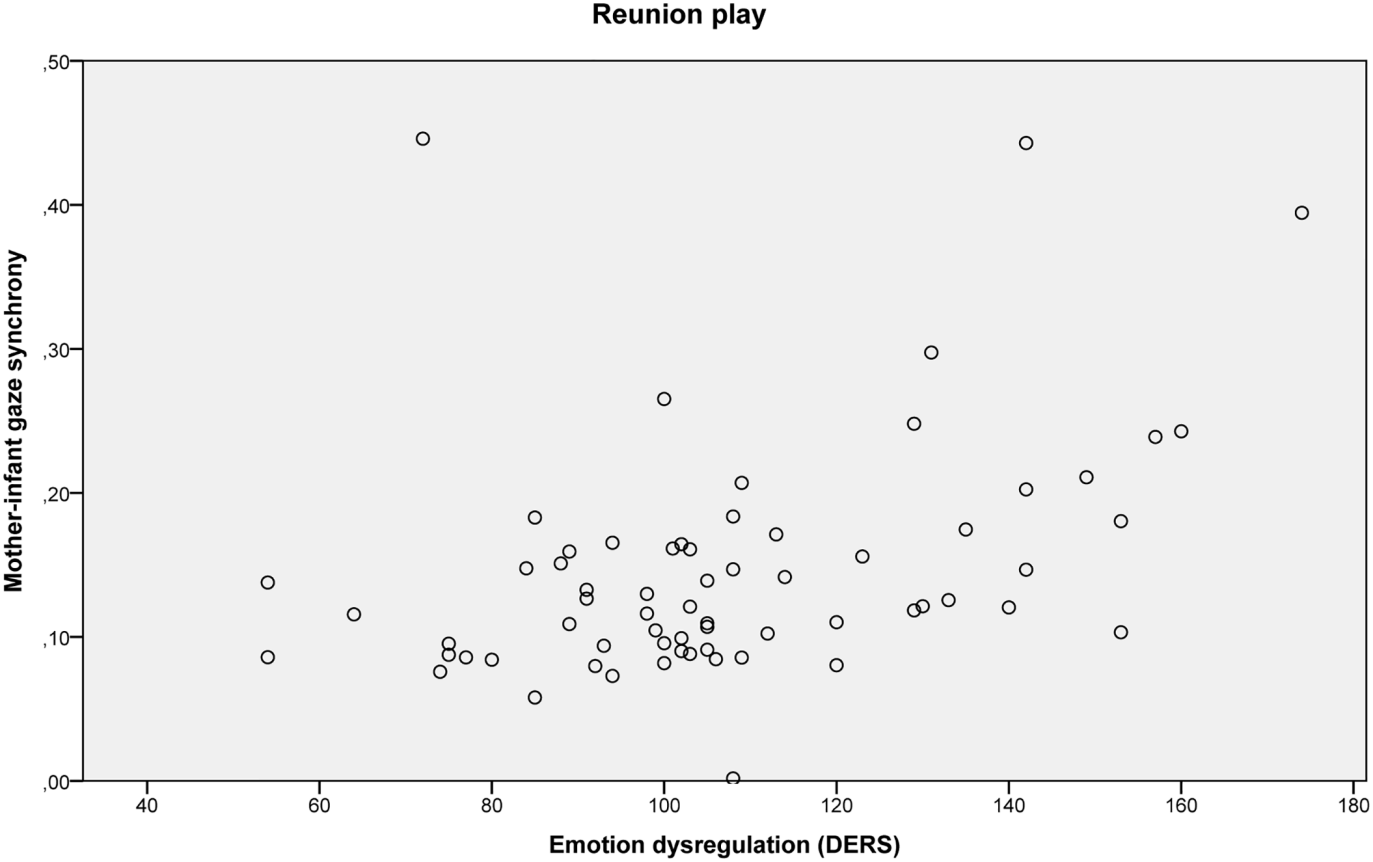
Scatter plot of the mothers’ emotion dysregulation scores and mother-infant gaze synchrony scores in the reunion play.

The scatter plot of the mothers’ emotion dysregulation scores versus mother-infant gaze synchrony scores in the reunion play are plotted in Fig 4.

Descriptively, higher maternal emotion dysregulation was related to higher mother-infant gaze synchrony in the initial and reunion play.

The fixed and random effect estimates for the predictors of mother-infant gaze synchrony are presented in Table 4.

**Table 4:** Fixed and Random Effects Estimates of the Predictors of Mother-Infant Gaze Synchrony (*N* = 68).

Parameter	Model 0		Model 1		Model 2		Model 3		Model 4	
	Effect	95% CI	Effect	95% CI	Effect	95% CI	Effect	95% CI	Effect	95% CI
Fixed effects										
Intercept	0.02	-0.19; 0.23	0.28*	0.03; 0.52	0.13	-0.17; 0.43	0.13	-0.16; 0.42	0.12	-0.17; 0.41
Level 1										
Initial play (vs. reunion play)			-0.51***	-0.77; -0.26	-0.51***	-0.77; -0.25	-0.51***	-0.76; -0.25	-0.49***	-0.74; -0.24
Level 2										
Infant female					0.31	-0.11; 0.72	0.28	-0.13; 0.68	0.28	-0.13; 0.69
Infant age					-0.01	-0.22; 0.20	-0.01	-0.22; 0.19	-0.01	-0.22; 0.20
Depressive symptoms					0.27*	0.03; 0.50	0.06	-0.25; 0.37	0.05	-0.25; 0.36
Anxiety symptoms					0.08	-0.16; 0.31	0.05	-0.18; 0.28	0.05	-0.18; 0.28
Emotion dysregulation							0.31*	0.01; 0.62	0.43*	0.09; 0.76
Cross-level interaction										
Emotion dysregulation*reunion play									-0.23	-0.49; 0.04
Random effects										
Level 1	0.66***	0.47; 0.93	0.54***	0.38; 0.76	0.54***	0.38; 0.76	0.54***	0.38; 0.76	0.53***	0.38; 0.75
Level 2	0.41**	0.21; 0.81	0.46***	0.26; 0.82	0.37**	0.19; 0.71	0.34**	0.17; 0.67	0.35**	0.18; 0.69
ICC	.38		.46		.41		.39		.40	
AIC	384.5		372.2		367.0		377.3		364.3	

Multilevel Random Coefficient Model. *N* = 136 play conditions nested in 68 mother-infant dyads. Level 1 predictor explains variance within dyads. Level 2 predictors explain variance between dyads. Cross-level interaction = interaction between Level 1 and Level 2. Fixed effects estimates are standardized. AIC = Akaike Information Criterion.

*p ≤ .05.

**p ≤ .01.

***p ≤ .001.

The random coefficient explained 37.8% of the variance of mother-infant gaze synchrony. The play condition was significantly related to mother-infant gaze synchrony; mothers and infants increased gaze synchrony in the reunion play compared with the initial play (Model 1). When the control variables (infant gender, infant age, maternal anxiety symptoms) and maternal depressive symptoms were entered into the model (Model 2), only maternal depressive symptoms were significantly positively associated with mother-infant gaze synchrony.

When maternal emotion dysregulation was added to the model (Model 3), maternal depressive symptoms were unrelated to mother-infant gaze synchrony. Instead, we found that maternal greater emotion dysregulation was related to higher mother-infant gaze synchrony.

Model 4 including all predictors (play condition, infant gender, infant age, maternal anxiety, maternal depressive symptoms, maternal emotion dysregulation, interaction term of maternal emotion dysregulation and play condition) showed the best model fit to the data (AIC = 364.3). Greater maternal emotion dysregulation was associated with higher mother-infant gaze synchrony. In contrast, infant gender, infant age, maternal depressive symptoms and maternal anxiety symptoms were unrelated to mother-infant gaze synchrony. The play condition did not significantly moderate the effect of maternal emotion dysregulation on mother-infant gaze synchrony.

Maternal emotion dysregulation was relatively more important than maternal depressive symptoms in predicting mother-infant gaze synchrony. The importance weights of the maternal emotion dysregulation (∑ω = 1.71) differed substantially from those of the maternal depressive symptoms (∑ω = 1.07).

The mediation analysis indicated that maternal emotion dysregulation fully mediated the effect of maternal depressive symptoms on mother-infant gaze synchrony. Using maternal emotion dysregulation as the criterion variable and maternal depressive symptoms as the predictor variable, maternal depressive symptoms were significantly positively related to maternal emotion dysregulation (condition I, β = 0.74, 95% CI [0.63, 0.84], *p* < .001). Using mother-infant gaze synchrony as the criterion variable and maternal depressive symptoms as the predictor variable, maternal depressive symptoms were significantly positively related to mother-infant gaze synchrony (condition II, β = 0.32, 95% CI [0.12, 0.51], *p* = .002). Using mother-infant gaze synchrony as the criterion variable and emotion dysregulation as predictor variable, emotion dysregulation was significantly related to mother-infant gaze synchrony (condition III, β = 0.40, 95% CI [0.20, 0.59], *p* < .001). Using mother-infant gaze synchrony as the criterion variable and maternal depressive symptoms and emotion dysregulation as predictors, maternal emotion dysregulation was significantly positively related to mother-infant gaze synchrony (β = 0.34, 95% CI [0.04, 0.65], *p* = .027), whereas maternal depressive symptoms were not significantly related to mother-infant gaze synchrony (condition IV, β = 0.07, 95% CI [-0.22, 0.36], *p* = .643).

Mothers more often synchronized with their infant’s behavior than infants synchronized with their mother’s behavior. In the initial play interaction, 46.4% of the synchronous interactions were caused by the mothers’ responses to their infant’s behavior; 28.6% of the synchronous interactions were caused by the infants’ responses to their mother’s behavior; and 25.0% of the synchronous interactions were driven by both interaction partners. In the reunion play, 71.4% of the synchronous interactions were caused by the mothers; 20.0% of the synchronous interactions were caused by the infants; and 8.6% of the synchronous interactions were driven by both interaction partners.

## Discussion

No earlier studies have tested the relationships between maternal emotion dysregulation and mother-infant gaze synchrony. We hypothesized that maternal self-reported emotion dysregulation is significantly related to mother-infant gaze synchrony. Our hypothesis was confirmed: we found that the mothers’ greater emotion dysregulation was significantly related to heightened mother-infant gaze synchrony in the initial and reunion play. Mother-infant gaze synchrony was most often produced by the mothers’ response to their infant’s gaze behavior. Mothers with greater emotion dysregulation may experience higher levels of distress and arousal [88]; higher distress may lead to heightened mother-infant gaze synchrony [34, 89, 90]. This heightened mother-infant gaze synchrony may interfere with the infant’s ability to self-regulate, to explore, and to respond adaptively to the environment [91, 92].

Mother-infant gaze synchrony increased from the initial play to the reunion play. In the reunion play, mothers and infants are faced with the challenge to reengage in a coordinated interaction after social stress had been introduced by the mother’s non-responsiveness [69]. Hence, the increased mother-infant gaze synchrony in the reunion play may indicate higher distress [69]. Consistent with this assumption, we observed increased distress indicated by the infants’ gaze aversion in the reunion play [93, 94].

We expected that the effect of maternal emotion dysregulation on mother-infant gaze synchrony would be more pronounced during the reunion play than during the initial play, assuming that the mothers’ emotion regulation abilities would be particularly challenged after the induction of distress by means of the still-face episode. We found that the effect of maternal emotion dysregulation on mother-infant gaze synchrony did not differ significantly between the initial and reunion play, although we found a trend in the expected direction. Thus, mothers with greater emotion dysregulation may display heightened mother-infant gaze synchrony in general rather than only in stressful interactions. Further studies have to clarify whether the effect of emotion dysregulation on mother-infant gaze synchrony is more pronounced in interactions with higher regulatory demands.

Maternal depressive symptoms were significantly related to mother-infant gaze synchrony, as long as we ignored the effect of maternal emotion dysregulation. This finding is consistent with earlier studies that reported both heightened [35] and lowered [33–35] mother-infant gaze synchrony in mothers with greater depression-related symptoms. A different picture emerged when we considered the effect of maternal emotion dysregulation on mother-infant gaze synchrony: maternal emotion dysregulation, but not maternal depressive symptoms, significantly predicted heightened mother-infant gaze synchrony. Furthermore, the overall effect of maternal emotion dysregulation on mother-infant gaze synchrony was relatively more important than the effect of maternal depressive symptoms in the five tested models. Our results indicate that the mothers’ emotion dysregulation may be an important predictor of deviant mother-infant gaze synchrony. Consequently, future studies should not only address the effect of maternal depressive symptoms on mother-infant gaze synchrony, but should additionally consider the effect of maternal emotion dysregulation on mother-infant gaze synchrony.

In our mediation analysis, we found that maternal emotion dysregulation fully mediated the effect of maternal depressive symptoms on mother-infant gaze synchrony. This result indicates that the association between maternal depressive symptoms and mother-infant gaze synchrony is explained through emotion dysregulation. Mothers with depressive symptoms but with strong emotion regulation abilities may be expected to be unimpaired in their mother-infant gaze synchrony. These results are consistent with a model of affective processes in parenting [95], which proposes that the ability to adaptively regulate emotions, rather than the experience of emotions per se, determine the quality of the parent-infant interaction.

Our study, using a sample of mothers with mood disorders, and previous studies, using non-clinical samples, reported that greater emotion dysregulation or depressive symptoms predicted lowered or heightened gaze synchrony [33–35]. These results converge with an “optimal midrange model” of interactive synchrony [11, 90, 96]: a well-organized and flexible interaction between mother and infant is driven by a moderate degree of gaze synchrony, whereas a heightened (extreme attentiveness to each other’s gaze) or a lowered degree of mother-infant gaze synchrony (reduced attentiveness to each other’s gaze) reflect interactive dysregulation.

Whether mother-infant gaze synchrony is either lowered or heightened might depend on the severity of the mother’s psychopathological symptoms. We observed severely mentally ill mothers diagnosed with a DSM-IV mood disorder. The majority of these mothers suffered from additional mental illnesses. The mothers likely experienced high levels of stress and arousal [97], which might trigger heightened responses to the infant’s gaze, thus leading to heightened mother-infant gaze synchrony. Most of the earlier studies reported that mother-infant gaze synchrony was lowered in dyads of mothers with greater depression-related symptoms compared with dyads of mothers with lower depression-related symptoms [33–35]. However, these studies used nonclinical mothers not diagnosed with a mood disorder, who were likely to behave differently than the mothers in our sample. Additional studies should clarify whether severe levels of depressive symptoms predict heightened mother-infant gaze synchrony, whereas sub-clinical levels of depressive symptoms predict lowered mother-infant gaze synchrony.

Alternatively, lowered and heightened mother-infant gaze synchrony may characterize the two sides of the same “coin” of disorganized parenting that is found in mothers with depression: withdrawing or intruding interactional behaviors (for reviews see [26, 27]). Withdrawn mothers might be characterized by lowered mother-infant gaze synchrony, whereas intrusive mothers might show heightened mother-infant gaze synchrony. Because greater emotion dysregulation was related to heightened mother-infant gaze synchrony in our study, the mothers with an intrusive parenting style may have dominated our sample.

In line with previous studies using nonclinical samples [8, 98–100], mothers spent a larger proportion of time in gazing at their infant’s face than infants gazed at their mother’s face. Mothers and infants gazed approximately five times per minute at the face of the interaction partner. In contrast, earlier studies using nonclinical samples reported lower gaze frequencies at the partner’s face, ranging between 1 and 3 times per minute in mothers [101–103] and infants [101, 103–105].

Maternal anxiety symptoms—about half of our mothers suffered from an additional anxiety disorder—were unrelated to mother-infant gaze synchrony. This counterintuitive result might be biased by the composition of our sample that included mothers who were diagnosed with mood disorders. The mothers with lower anxiety symptoms suffered from depressive symptoms that might have obscured the impact of anxiety symptoms on mother-infant gaze synchrony. However, two earlier studies concurred with our finding: the severity of the maternal anxiety symptoms [82] or the diagnosis of an anxiety disorder [22] did not predict mother-infant synchrony. Additional studies should consolidate these findings.

We controlled our analysis for infant age and gender and found that these infant characteristics were unrelated to mother-infant gaze synchrony. Earlier studies on mother-infant gaze synchrony also reported no effects of infant age or infant gender on mother-infant gaze synchrony [33–35]. Additional research might clarify whether mother-infant gaze synchrony is invariant across infant gender and infant age.

Our study has strengths and weaknesses that the readers should note when interpreting our results. We included mothers with mood disorders that varied considerably in self-reported emotion dysregulation and depressive symptoms, thus strengthening the validity of our results. A weakness of our study is that we assessed emotion dysregulation and depressive symptoms by maternal self-report. The mothers’ self-reported depressive symptoms and emotion dysregulation may differ from systematic measurements conducted by an interviewer or an observer. Our study also missed a control group of mentally healthy mothers, limiting the generalizability of our findings to mothers with mood disorders. Additional studies should compare the effects of emotion dysregulation and depressive symptoms on mother-infant gaze synchrony between mothers with and without mood disorders. Studies that include mothers with both sub-clinical and clinical levels of depressive symptoms might also be helpful because they might identify a non-linear relationship between depressive symptoms and mother-infant gaze synchrony: sub-clinical levels of depressive symptoms might be related to lowered mother-infant gaze synchrony, whereas more severe levels of depressive symptoms might be related to heightened mother-infant gaze synchrony. It might be also valuable to explore the effects of different subtypes of emotion dysregulation on mother-infant gaze synchrony.

The readers of this study might also note that about half of the mothers with mood disorders suffered from a co-morbid anxiety disorder. Multiple diagnoses of mental disorders are the norm, rather than the exception, in individuals diagnosed with a mental disorder [106], which makes it difficult to disentangle the effects of overlapping symptoms on mother-infant gaze synchrony. We addressed this problem by controlling our statistical analysis for anxiety symptoms; nevertheless, mothers with a single diagnosis of depression might show different forms of emotion dysregulation than mothers with co-morbid anxiety disorders. Additional research that compares mothers with a single diagnosis of a mood disorder with mothers with a co-morbid diagnosis of an anxiety disorder may clarify the specificity of our findings. The recruitment of patients from a psychiatric mother-infant unit may have biased the selection of the study participants. Our sample included severely mentally ill mothers, and might not be representative for mothers with less severe mood disorders. Finally, the variation of the infants’ ages limits our study, which might have caused differences in the mothers’ and infants’ gaze behavior. However, we statistically controlled our analysis for infant age and found that infant age was unrelated to mother-infant gaze synchrony.

Our findings suggest steps for improving future research. First, future studies on mother-infant gaze synchrony should consider not only maternal depressive symptoms but also maternal emotion dysregulation. These studies might longitudinally assess maternal emotion dysregulation and mother-infant gaze synchrony to examine whether maternal emotion dysregulation temporarily precedes mother-infant gaze synchrony. It might also be important to study whether changes in maternal emotion dysregulation, modified by an emotion regulation-focused intervention [107, 108], would correspond with changes in mother-infant gaze synchrony. Future studies should consider the consequences of lowered and heightened mother-infant gaze synchrony on the infant’s development.

Mother-infant synchrony, which is the fine-grained interactive temporal coordination of behaviors between mother and infant, provides the foundation for the infant’s self-regulation, attachment and cognitive abilities [16, 24, 36]. We should understand which factors disrupt mother-infant synchrony. We could then target these factors by interventions for mothers with postpartum mental disorders and their infants to promote mental health in the mother and the child. Our study identified the mother’s emotion dysregulation as a risk factor for heightened mother-infant gaze synchrony, which was overlooked in earlier research. The ability to *regulate* negative emotions, for example, sadness in depression, might be a key ability for the interactive regulation of gaze between mother and infant. If further studies replicate our study results, clinicians should focus on maternal emotion dysregulation. For example, mother-infant treatment could be complemented by an intervention that targets maternal emotion-regulation abilities [109–111]. Clinicians may also intervene with video feedback techniques that focus on the mother’s heightened reaction by helping the mother to more clearly read and understand her infant’s gaze signals and to be less reactive to her infant’s gaze.

## Conclusions

We found that maternal-reported emotion dysregulation in mothers with mood disorders significantly heightened mother-infant gaze synchrony. Maternal self-reported depressive symptoms were less important than maternal emotion dysregulation in predicting mother-infant gaze synchrony. Furthermore, maternal emotion dysregulation fully mediated the effect of maternal depressive symptoms on mother-infant gaze synchrony. Our results indicate that the mother’s emotion dysregulation matters for mother-infant gaze synchrony between mothers with mood disorders and their infants—not only the mother’s depressive symptoms. Moreover, maternal emotion dysregulation may be a mechanism underlying the association between maternal depressive symptoms and mother-infant gaze synchrony. More evidence on the relations between emotion dysregulation, depressive symptoms and mother-infant gaze synchrony is needed.
